# Vitamin D in Primary Sjogren’s Syndrome (pSS) and the Identification of Novel Single-Nucleotide Polymorphisms Involved in the Development of pSS-Associated Diseases

**DOI:** 10.3390/diagnostics14182035

**Published:** 2024-09-13

**Authors:** Siarhei A. Dabravolski, Alexey V. Churov, Irina A. Starodubtseva, Dmitry F. Beloyartsev, Tatiana I. Kovyanova, Vasily N. Sukhorukov, Nikolay A. Orekhov

**Affiliations:** 1 Department of Biotechnology Engineering, Braude Academic College of Engineering, Snunit 51, Karmiel 2161002, Israel; 2 Institute of General Pathology and Pathophysiology, 8 Baltiyskaya Street, 125315 Moscow, Russia; achurou@yandex.ru (A.V.C.); kovyanovat@gmail.com (T.I.K.); vnsukhorukov@gmail.com (V.N.S.); www.fuper@gmail.com (N.A.O.); 3 Institute on Aging Research, Russian Gerontology Clinical Research Center, Pirogov Russian National Research Medical University, 16 1st Leonova Street, 129226 Moscow, Russia; 4 Department of Polyclinic Therapy, NN Burdenko Voronezh State Medical University, 10 Studencheskaya Street, 394036 Voronezh, Russia; starodubtsevairina1@gmail.com; 5 Vascular Surgery Department, A. V. Vishnevsky National Medical Research Center of Surgery, 27 Bolshaya Serpukhovskaya Street, 117997 Moscow, Russia; beloyar@rambler.ru; 6 Institute for Atherosclerosis Research, Osennyaya Street 4-1-207, 121609 Moscow, Russia

**Keywords:** autoimmunity, Sjögren’s syndrome, single-nucleotide polymorphisms, GWASs, lymphoma, fatigue

## Abstract

Sjögren’s syndrome (SS) is a chronic autoimmune disorder characterised by lymphocytic infiltration of the exocrine glands, which leads to dryness of the eyes and mouth; systemic manifestations such as arthritis, vasculitis, and interstitial lung disease; and increased risks of lymphoma and cardiovascular diseases. SS predominantly affects women, with a strong genetic component linked to sex chromosomes. Genome-wide association studies (GWASs) have identified numerous single-nucleotide polymorphisms (SNPs) associated with primary SS (pSS), revealing insights into its pathogenesis. The adaptive and innate immune systems are crucial to SS’s development, with viral infections implicated as environmental triggers that exacerbate autoimmune responses in genetically susceptible individuals. Moreover, recent research has highlighted the role of vitamin D in modulating immune responses in pSS patients, suggesting its potential therapeutic implications. In this review, we focus on the recently identified SNPs in genes like OAS1, NUDT15, LINC00243, TNXB, and THBS1, which have been associated with increased risks of developing more severe symptoms and other diseases such as fatigue, lymphoma, neuromyelitis optica spectrum disorder (NMOSD), dry eye syndrome (DES), and adverse drug reactions. Future studies should focus on larger, multi-ethnic cohorts with standardised protocols to validate findings and identify new associations. Integrating genetic testing into clinical practise holds promise for improving SS management and treatment strategies, enabling personalised interventions based on comprehensive genetic profiles. By focusing on specific SNPs, vitamin D, and their implications, future research can lead to more effective and personalised approaches for managing pSS and its complications.

## 1. Introduction

Sjögren’s syndrome (SS) is a chronic autoimmune disorder characterised by lymphocytic infiltration of the exocrine glands, leading to dryness of the eyes and mouth, and it is often accompanied by systemic manifestations affecting various tissues and organs, such as arthritis, vasculitis, and interstitial lung disease, and an increased risk of lymphoma, cardiovascular diseases, and cancers [1,2,3,4,5,6]. The clinical spectrum of SS is broad, encompassing both primary SS (pSS), in which it occurs alone, and secondary SS (sSS), which develops alongside other autoimmune conditions such as rheumatoid arthritis (RA) or systemic lupus erythematosus (SLE) [7]. This debilitating condition primarily affects women, with a striking female to male ratio ranging from 9:1 to 14:1 [8]. SS is one of the most common autoimmune diseases, with an estimated global prevalence of 0.1% to 4.8%, depending on the population studied and diagnostic criteria used [9,10].

The pronounced female predominance of SS suggests a strong genetic component linked to sex chromosomes [11]. Notably, individuals with Klinefelter’s syndrome (47,XXY), who have an extra X chromosome, exhibit a risk profile similar to females (46,XX), highlighting how the dosage of X chromosome genes affects SS susceptibility [12]. Conversely, the coexistence of Turner syndrome (45,X) with SS is exceedingly rare, showing that the SS risk in these patients is similar to that of 46,XY males rather than that of 46,XX females [13]. Structural chromosomal aberrations affecting X chromosome regions have been identified in SS patients, suggesting the presence of undefined dosage-sensitive risk genes within these loci [14]. Another possible mechanism underlying the effects of the X chromosome dose in pSS may be based on the genes escaping X inactivation. As we know, one female X chromosome gene randomly undergoes X-inactivation to achieve equal gene expression between XY males and XX females. However, up to 15% of X genes may escape that X-inactivation [15], thus increasing the gene dose and predisposing women and men with more than one X chromosome to differential expression of these genes. Furthermore, among many single-nucleotide polymorphisms (SNPs), which have been identified through genome-wide association studies (GWASs) and associated with pSS, some SNPs were believed to act in a sex-specific way. There is no difference in the frequency of pSS-associated SNPs between women and men in the general population, but there is a much higher likelihood of pSS development in women carrying certain SNPs, which can affect the expression of genes in pSS-associated regions [16,17]. 

*Human leukocyte antigen* (*HLA*) genes, particularly *HLA* class II genes (such as *HLA-DR* and *HLA-DQ*), play a critical role in SS pathogenesis by presenting self-antigens to T cells and triggering autoantibody production [18]. Specific *HLA* alleles, including HLA-DRB1*15:01 and HLA-DRB1*03:01, have consistently been associated with an increased risk of SS across different populations [19,20,21]. These alleles contribute to the formation of autoantibodies against Ro (SSA) and La (SSB) antigens, which are serological hallmarks of the disease and are important for clinical diagnosis [22].

Dysregulation of both adaptive and innate immune responses is central to SS’s pathogenesis. The adaptive immune system, comprising B and T lymphocytes, plays a pivotal role in SS through aberrant activation and differentiation [23]. CD4+ T cells, particularly Th1 and Th17 subsets, are prominently involved in the inflammatory cascade, secreting pro-inflammatory cytokines such as interleukin-17 (IL-17) and interferon-gamma (IFNγ) that contribute to tissue damage [24]. B cells in pSS patients produce autoantibodies that target nuclear antigens, further perpetuating immune-mediated destruction of exocrine glands and systemic inflammation [25]. Innate immune responses, mediated by pattern recognition receptors (PRRs) such as Toll-like receptors (TLRs), initiate the inflammatory cascade upon recognition of microbial components or endogenous danger signals [26,27]. Viral infections, particularly those with tropism for salivary and lacrimal glands, have been implicated as environmental triggers in pSS, inducing chronic activation of innate immune pathways and exacerbating autoimmune responses [28]. This sustained inflammatory milieu perpetuates glandular destruction and amplifies systemic manifestations in susceptible individuals [29].

Recent advancements in genomic technologies have facilitated the identification of multiple SNPs associated with SS susceptibility. These SNPs often reside within genes that are involved in immune regulation, cellular signalling pathways, and cytokine production, offering mechanistic insights into disease pathogenesis [20,21]. For instance, the association with pSS susceptibility was defined in recent meta-analysis papers for a low copy number of *Fc Fragment Of IgG Receptor IIIb* (*FCGR3B*) [30], *family with sequence similarity 167A-B lymphoid tyrosine kinase* (*FAM167A-BLK*) rs2736340 T allele [31], *Tumour necrosis factor superfamily member 4* (*TNFSF4*) rs2205960 G>A [32], *Neutrophil Cytosolic Factor 1* (*NCF1*) rs201802880 (Arg90His hypoactive variant) [33], CGGGG indel polymorphism in the *Interferon Regulatory Factor 5* (*IRF5*) [34], and *B cell activating factor* (*BAFF*) (rs12583006 T>A) AA genotype [35].

Besides genetic factors, the involvement of epigenetic mechanisms, such as DNA methylation, histone modifications and non-coding RNAs, has been extensively studied, and antigen presentation, lymphocyte regulation, Toll-like receptor, and interferon signalling have been suggested as the most relevant therapeutic targets. These topics were recently reviewed in some excellent published papers and will be omitted here [22,36].

Despite the significant progress that has been made in understanding the genetic and immunological underpinnings of pSS, challenges remain when it comes to translating these insights into clinical practice. The clinical heterogeneity of SS necessitates personalised approaches to its diagnosis, management, and treatment [37]. Biomarkers derived from genetic and omics studies, including SNPs associated with disease susceptibility, hold promise for improving diagnostic accuracy and predicting disease progression in pSS patients. Thus, increased signalling potentials in peripheral B cells through STAT3 S727 and NF-κB pathways were found to correlate with a type I IFN signature of pSS patients, suggesting that there are significant potential benefits of therapies targeting these pathways [38]. In another study, while anti-Ro/SSA and anti-La/SSB were present in all pSS patients, three distinctive molecular phenotypes were identified: (1) no significant elevation of IFN or inflammation modules; (2) strong IFN and inflammation modular network signatures and high plasma protein levels of CXC Motif Chemokine Ligand 9 and 10 (CXCL9/10), BAFF and tumour necrosis factor superfamily member 14 (TNFSF14 or LIGHT); and 3) moderately elevated IFN modules, suppressed inflammatory modules, and increased plasma levels of CXCL9/10/13, IL-1a, and IL-21 [39]. 

On the contrary, two distinct subtypes of pSS have been identified in another study, which was based on the presence/absence of SSA/SSB antibodies and SNPs. Therefore, the first high-pSS-risk group was associated with the presence of SSA/SSB antibodies and *HLA-DQA1* rs6933289, *IRF5* rs3823536, and *Glutamic-Oxaloacetic Transaminase 1* (*GOT1*) rs4919321. Furthermore, the patients of this group displayed a more severe disease phenotype (increased prevalence of purpura, major salivary gland swelling, lymphadenopathy, and lymphoma) compared to the second group, which were SSA-/SSB-negative and had no associated SNPs [40]. Finally, INF signatures, SNPs, methylation, flow cytometry analysis of alterations in patterns of peripheral blood leukocytes, cytokine analysis, and clinical parameters were used in a large cross-sectional cohort study to identify four groups of patients with distinct patterns of immune dysregulation. Therefore, 35 SNPs were detected in group 1, 6 SNPs were detected in group 3, only 1 SNP was detected in group 4, and no SNPs were detected in group 2. Unsurprisingly, most of the SNPs were associated with either the immune system (*HLA-DQB1*, *HLA-DQA1*, *HLA-DRA*, *HLA-C*, *HLA-G*, *Butyrophilin Like 2* (*BTNL2*), *HLA Complex Group 23* (*HCG23*)), signal transduction (*Neurogenic Locus Notch Homolog Protein 4* (*NOTCH4*), developmental biology (*POU Class 5 Homeobox 1* (*POU5F1*), gene expression (*DExD-Box Helicase 39B* (*DDX39B*) or the cell cycle (*Tubulin Beta Class I* (*TUBB*) [41]. Overall, a clear understanding of pSS heterogeneity providing clinically and immunopathologically relevant signatures would help to develop precise and personalised treatment strategies.

This review aims to synthesise recent findings on the role of vitamin D in pSS and novel SNPs associated with pSS patients’ susceptibility to more severe pSS symptoms and to other diseases (most importantly, lymphoma and fatigue), offering a detailed overview of the genetic risk factors and their implications for disease pathogenesis. Because the association between the *HLA*-region SNPs and pSS was extensively covered in several recent studies [18,20,21,22], we will omit it here. By elucidating the complex interplay between genetic predisposition, immune dysregulation, and environmental triggers, this review seeks to provide insights into potential therapeutic targets and personalised medicine strategies for pSS and associated diseases.

## 2. Immunomodulatory Function of Vitamin D

**Figure 1:**
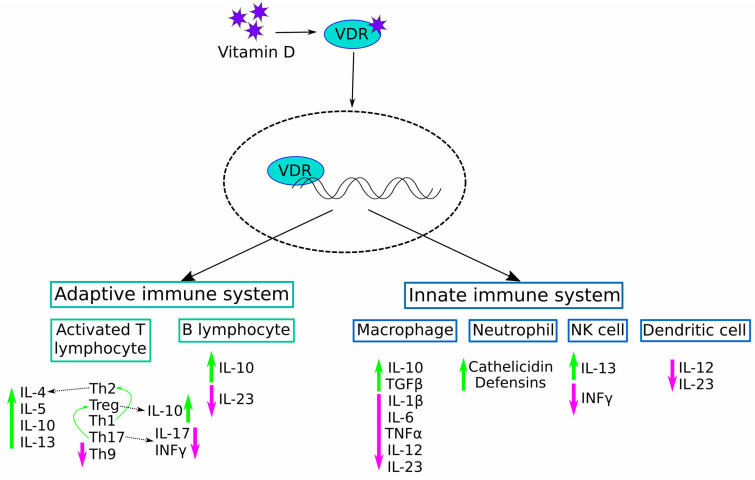
Immunomodulatory effects of vitamin D on innate and adaptive immune responses. The figure describes the effect of vitamin D on B lymphocytes and activated T lymphocytes (the adaptive immune system) and macrophages, neutrophils, NKs, and dendritic cells (the innate immune system). Vitamin D drives the phenotype shift from Th17 and Th1 to Treg and Th2, respectively. Accordingly, the production of INFγ and IL-17 by Th17 is decreased, while the production of IL-10 and IL-4/5/10/13 by Treg and Th2, respectively, is increased. The green and magenta arrows represent, respectively, the increase and decrease in the production of designated molecule.

Vitamin D, a steroid hormone vital to calcium homeostasis and bone health, has emerged as a pivotal regulator of the immune system. Beyond its classical role in skeletal integrity, extensive research over recent decades has illuminated its potent immunomodulatory properties, implicating it in various facets of immune function and autoimmune diseases [42,43]. It was observed to stimulate immune responses, particularly against infectious pathogens like tuberculosis and leprosy [44,45]. These findings marked the early understanding of vitamin D not just as a nutritional factor but also as a hormone with profound effects on the immune system. In particular, vitamin D exerts its immunomodulatory effects through several mechanisms involving both innate and adaptive immune responses (Figure 1). At the level of innate immunity, vitamin D plays a crucial role in enhancing antimicrobial peptide production in epithelial cells, such as defensin β2 and cathelicidin, which are pivotal in innate defence against invading pathogens [46]. In the skin, for instance, where vitamin D is synthesised in response to exposure to sunlight, it promotes the integrity of the epithelial barrier and assists with local immune responses [47]. Moreover, vitamin D regulates the function of macrophages and monocytes, which are key players in the innate immune system. These cells express *vitamin D receptors* (*VDRs*) and can produce the active form of vitamin D (1,25-dihydroxyvitamin D3 or 1,25(OH)2D3) locally in response to infections [48]. 1,25(OH)2D3 enhances the innate immune response by promoting phagocytosis, cytokine production, and antimicrobial peptide synthesis, contributing to pathogen clearance and immune surveillance [49].

In adaptive immunity, vitamin D plays a pivotal role in modulating the functions of T cells, B cells, and dendritic cells (DCs). T regulatory cells (Tregs), a subset of T cells crucial for maintaining immune tolerance and preventing autoimmunity, are particularly influenced by vitamin D [50,51]. Vitamin D promotes the development and function of Tregs, thereby suppressing excessive immune responses and promoting immune homeostasis [52]. Furthermore, vitamin D influences antigen presentation and cytokine production by dendritic cells, and these processes are pivotal in activating T cells and shaping immune responses [53]. By promoting an anti-inflammatory cytokine profile and dampening pro-inflammatory responses, vitamin D helps maintain immune balance and tolerance, which are crucial for preventing autoimmune reactions (Figure 1) [54].

The relationship between vitamin D deficiency and autoimmune diseases has garnered significant attention in epidemiological studies worldwide. Numerous investigations have highlighted a consistent association between low vitamin D levels and increased risk or severity of autoimmune conditions [55]. Rheumatoid arthritis (RA), for instance, exhibits higher prevalence and disease activity in individuals with deficient vitamin D status [56,57]. Similar associations have been observed in systemic lupus erythematosus (SLE) [58], multiple sclerosis (MS) [59,60], and inflammatory bowel disease (IBD) [61], among others [62,63].

The mechanisms underlying vitamin D’s protective role in autoimmune diseases are multifaceted. Vitamin D deficiency may exacerbate autoimmune responses by impairing immune regulation and promoting pro-inflammatory pathways [64,65]. Conversely, vitamin D supplementation has shown promising immunomodulatory effects with regard to mitigating disease activity and improving clinical outcomes in autoimmune patients. Clinical trials and observational studies have explored the efficacy of vitamin D supplementation in autoimmune conditions, aiming to optimise treatment outcomes and improve patients’ quality of life [66,67,68,69]. However, challenges remain, including variability in individual responses to vitamin D therapy and the optimal dosing regimens required to achieve therapeutic benefits. Future research directions include elucidating the molecular mechanisms underlying vitamin D’s immunomodulatory effects, exploring its interactions with other immune regulatory pathways, and conducting robust clinical trials to establish evidence-based guidelines for vitamin D supplementation in autoimmune disease management.

### The Role of Vitamin D in pSS

The association between vitamin D and pSS is rather contradictory. Thus, low levels of vitamin D were found to be associated with pSS in some meta-analyses [70,71], while no association was found in another study [72]. Moreover, a low serum level of Calcifediol (25(OH)D3) was associated with the corneal staining score, conjunctival staining score, Schirmer I value, and tear breakup time in Korean pSS patients [73]. Similarly, another Korean study found that low serum levels of 25(OH)-D3 were associated with EULAR Sjogren’s syndrome disease activity index in pSS patients [74]. On the contrary, no association was found in Indian [75] and Chinese [76] pSS patients. These results suggested a strong need for studies with larger sample sizes to further unravel the potential causal relationship and the exact underlying molecular mechanisms.

Recent experiments on pSS mice model (thrombospondin-1 knock-out (TSP-1 KO) mice) and peripheral blood mononuclear cells (PBMCs) derived from pSS patients provided some novel insights into the functional significance of vitamin D in pSS. Therefore, TSP-1 KO mice demonstrated reduced serum levels of 25(OH)D3 and developed dry eye, while oral vitamin D supplementation improved its serum levels and restored central corneal epithelial thickness and the corneal structure in a dose-dependent manner. Furthermore, 25(OH)D3 supplementation reduced corneal levels of TNF-Alpha-Converting Enzyme (TACE), TNFα, TGFα, VEGFR2, VEGFA, TGFβ, and SMAD2/3, thus inhibiting corneal neovascularisation, inflammation, derangement, and fibrosis [77]. Similarly, 25(OH)D3 treatment decreased Th lymphocyte proliferative responses and B lymphocyte proliferation in Turkish pSS patients and pSS PBMC cultures compared to PBMC cultures alone. Also, 25(OH)D3 treatment increased the frequency of *FoxP3* expressing CD4+CD25+ Treg cell, thus decreasing IFNγ and IL-17 secretion, while the level of IL-10 was increased (Figure 1). Finally, 25(OH)D3 treatment decreased plasma B cell subsets and increased the total memory B lymphocyte ratio in pSS patients compared to PBMC cultures alone [78]. Overall, these results proposed the anti-inflammatory and immunomodulatory effects of vitamin D supplementation as the main beneficial mechanisms of pSS treatment.

Interestingly, so far only one vitamin D-related SNP was characterised as a risk factor for pSS, SLE, and RA. Therefore, an analysis of Italian patients with autoimmune diseases showed that *vitamin D receptor* (*VDR*) rs7975232 (T>G) homozygous genotype (GG) was associated with susceptibility to all investigated autoimmune connective tissue disorders. Mechanically, rs7975232 GG was associated with production of anti-DNA, anti-RNP, anti-SSA, and anti-SSB autoantibodies in SLE patients, and with production of anti-SSA in pSS patients [79]. On the contrary, the VDR-focused investigation on the Hungarian population found no difference in *VDR* SNPs between pSS patients and healthy controls [80].

In total, the provided results suggest a rather contradictory role of vitamin D and *VDR* SNPs in pSS. However, it is important to note that while the role of vitamin D levels in pSS has been investigated in patients of many ethnicities, the number of studies focused on the identification of pSS-related SNPs in vitamin D metabolism genes is extremely limited. Therefore, the relationship between *VDR* SNPs rs7975232 and pSS may be specific only to the Italian population. Future multi-ethnic studies with larger sample sizes should be conducted to provide a clear view of the connection between vitamin D and the risk of pSS development throughout different populations.

## 3. SNPs Associated with Sjögren’s Syndrome

### 3.1. Overview of Genetic Findings in Sjögren’s Syndrome

In recent decade, implementation of next generation sequencing, novel bioinformatic and biostatistic approaches enabled specific genetic analyses of race-dependent pSS-specific SNPs. Such genome-wide association studies (GWASs) have been conducted on thousands of subjects from Europe [81,82], Italy [83,84], China [85,86], Korea [87], Asia [88], and others. As expected, some gene variants were significant in Europeans, but not in Chinese patients, suggesting that different populations may have different pSS risk-associated SNPs. The ‘classical’ PCR methods, expensive and time consuming, allowed to analyse very limited number of SNPs, while modern next-generation sequencing revealed tens of new SNPs. Therefore, the exploration of genetic underpinnings in pSS has unveiled significant insights into its pathogenesis, particularly through the study of specific gene variants and their role in disease susceptibility and phenotype variation. Further, we discuss cumulative findings from multiple studies (cited at the beginning of this paragraph) on genetic associations in pSS, emphasising inflammation and interferon signalling pathways, among others, and exploring ethnic differences in genetic susceptibility.

### 3.2. Key Genetic Pathways and Variants

Interferon pathway: The interferon (IFN) signalling pathway is a prominent feature in autoimmune diseases, including pSS. For example, the association of *Signal Transducer And Activator Of Transcription 4* (*STAT4*), a transcription factor that regulates T-helper differentiation and interferon production [89,90], with pSS, particularly the rs7574865 polymorphism, underscores the critical role of this pathway. The minor allele of rs7574865 has been linked to increased *STAT4* expression and heightened sensitivity to IFNα, which could enhance the autoimmune process in pSS patients. This association aligns with observations related to other autoimmune disorders like RA and SLE, where *STAT4* variants have been similarly implicated. Among other variants relevant for this pathway, we can also mention *Interferon Gamma Receptor* (*IFNGR1/2*), *Interferon Regulatory Factor 5* (*IRF5*), *Dihydrouridine Synthase 2* (*DUS2*).

Inflammatory pathways: Many studies have identified several genetic variants associated with inflammatory processes in pSS. Notably, the IL-10 gene, which encodes a cytokine that modulates inflammation and immune responses [91], exhibits dual effects in pSS. The rs3024505 variant is associated with increased susceptibility to SS, while the rs1800872 variant appears protective. IL-10’s role in regulating Th1 responses and modulating MHC class II antigens highlights its complex involvement in SS pathology. Variants of IL-10 could influence disease severity and progression by altering cytokine production and inflammatory responses. The findings related to the *HCP5* gene further illustrate the connection between genetic variants and inflammatory disease mechanisms. The rs3099844 variant in *HCP5* is linked to the presence of anti-SSB antibodies and autoantibodies such as rheumatoid factor (RF), suggesting that it plays a role in promoting a more aggressive inflammatory phenotype. *HCP5*’s association with autoantibody production, particularly in the context of pSS, highlights its potential role in disease severity and progression. The other genes related to this pathway are *Nuclear Factor NF-Kappa-B Activator 1* (*TRAF3IP2*), *TNF Alpha Induced Protein 3* (*TNFAIP3*), *IL-4R*, *IL-6*, *IL-12A*, *IL-12RB1*, *IL-12RB2*, *IL-18*, and *IL-18R1* [83].

T cell receptor (TCR) signalling pathway: Genetic studies have also pointed to the T cell pathway’s involvement in pSS. The discovery of common variants in TCR-pathway genes across multiple autoimmune diseases, including SS, RA, and SLE, suggests that T cell activation and signalling play a crucial role in autoimmune pathogenesis. Variants of genes like *Protein Tyrosine Phosphatase Receptor Type C* (*PTPRC*), *Lymphocyte Cell-Specific Protein-Tyrosine Kinase* (*LCK*), *Linker For Activation Of T Cells* (*LAT*), *Tec Protein Tyrosine Kinase* (*TEC*), and *Nuclear Factor Of Activated T Cells 5* (*NFAT5*), which are involved in TCR signalling, have been associated with susceptibility to autoimmune diseases. These findings support the hypothesis that T cell-driven immunity is a key factor in the development of autoimmune conditions, including pSS [92,93].

### 3.3. Ethnic Differences and Genetic Associations

A significant aspect of the genetic studies on pSS is the variation in genetic associations across different ethnic groups [82,86,87]. Thus, the international multi-ethnic GWASs highlighted distinct genetic associations in European and Asian populations, reflecting the diversity in genetic susceptibility [88]. For example, the association between *KLRG1* and SS was observed specifically in Asian cohorts, while European studies identified significant associations with different MHC regions. This variation underscores the importance of considering ethnic differences in genetic research and highlights the need for larger, more inclusive studies to capture the full spectrum of genetic diversity in autoimmune diseases.

The presence of SNPs with varying effects across ethnic groups suggests that genetic susceptibility to SS is influenced by population-specific allele frequencies and haplotype structures. Such differences can affect the interpretation of genetic findings and their application to clinical practise. Future research should aim to integrate genetic data from diverse populations to enhance our understanding of SS’s susceptibility and pathogenesis.

## 4. Interaction with Other Diseases

**Figure 2:**
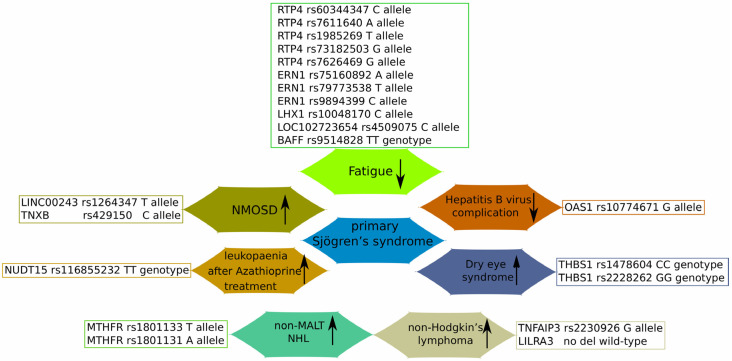
The role of SNPs in the development of Sjogren’s syndrome-associated diseases. Up- and downward arrows represent the increased/decreased risk of the described SNPs with regard to the development of particular pSS-associated diseases or complications. *Oligoadenylate synthetase 1* (*OAS1*); *receptor transporter protein 4* (*RTP4)*; *Endoplasmic reticulum to nucleus signalling 1* (*ERN1*); *LIM homeobox 1 locus* (*LHX1*); *B lymphocyte hyperactivity with B cell activating factor* (*BAFF*); *Tenascin XB* (*TNXB*); *Nudix Hydrolase 15* (*NUDT15*); *methylene tetrahydrofolate reductase* (*MTHFR*); *TNF Alpha-Induced Protein 3* (*TNFAIP3*); *Leukocyte immunoglobulin-like receptor A3* (*LILRA3*); *Thrombospondin 1* (*THBS1*).

Interestingly, previous findings showed an association between pSS and other diseases, suggesting the existence of shared genetic susceptibility loci involved in the co-occurrence of these diseases. For example, meta-analyses found that pSS-associated SNPs increased the primary biliary cholangitis, and vice versa, in a European population [94], while no impact on the risk of Parkinson’s Disease was found [95]. Moreover, a number of recent studies have investigated the impact of pSS-associated SNPs on the risk of developing other diseases (Table 1) (Figure 2).

The rs10774671 minor allele G of *2′–5′ oligoadenylate synthetase* (*OAS1*), a crucial interferon-induced gene involved in cell growth, apoptosis and anti-virus defence, was associated with a decreased risk of pSS, anti-SSA-positive pSS, and anti-SSA-positive pSS complicated with HBV infection, but did not decrease the risk of anti-SSA-negative pSS. Mechanically, A allele reduced the expression of the primary *p46* isoform of *OAS1*, while it increased the expression of three other isoforms (*p42*, *p44*, and *p48*), which are less effective in HBV replication inhibition. These results suggested that OAS1 rs10774671 A allele carriers had a shared susceptibility to HBV infection and pSS development [96].

A recent case study reported the development of severe leukopenia after the application of an immunosuppressive medication, Azathioprine (AZA), for pSS treatment. Further analysis revealed that the patient’s *Nudix Hydrolase 15* (*NUDT15*), which is responsible for hydrolysis of nucleoside diphosphates, contained an R139C mutation, which decreased the patient’s enzyme function. Apparently, the R139C version of *NUDT15* could not metabolise AZA with the necessary speed and caused an accumulation of 6-thioguanine nucleotides (6-TGN) in the erythrocytes. Therefore, mutant *NUDT15* R139C (TT genotype) and blood 6-TGN levels should be monitored in patients receiving AZA to predict and prevent AZA-induced myelosuppression [97].

Another study found a casual association between pSS and susceptibility to neuromyelitis optica spectrum disorder (NMOSD), another autoimmune demyelinating disease of the central nervous system, which may lead to blindness and paralysis. In particular, analysing GWAS results using the Mendelian randomization (MR) method showed that the rs1264347 T allele of *Long Intergenic Non-Protein Coding RNA 243* (*LINC00243*) and the rs429150 C allele of *Tenascin XB* (*TNXB*) increase the risk of developing NMOSD [98]. Moreover, previous studies demonstrated that *LINC00243* variants were involved in immune dysregulation associated with Type 1 diabetes [107], while *TNXB* variants can cause hereditary primary vesicoureteral reflux. However, prospective cohorts with large sample sizes and further lab experiments may be required to evaluate the exact role of *LINC00243* and *TNXB* SNPs in pSS and NMOSD [108]. Furthermore, the development of dry eye syndrome (DES), an ophthalmological complication of SS that manifests in the form of corneal nerve fibre lesions, was recently associated with SNPs in *Thrombospondin 1* (*THBS1*). Therefore, rs1478604 and rs2228262 *THBS1* SNPs correlated with coefficients of anisotropy and symmetry, predisposing the rs1478604 CC genotype and rs2228262 GG with DES severity [106].

Fatigue, often described as ‘an overwhelming sense of tiredness, exhaustion, and lack of energy’, affects the majority of pSS patients and greatly reduces their quality of life [109]. Recently, several SNPs have been linked to fatigue in pSS, including several SNPs between *receptor transporter protein 4/Mannan-binding lectin serine peptidase 1* (*RTP4/MASP1*), *endoplasmic reticulum to nucleus signalling 1* (*ERN1*), *LIM homeobox 1 locus* (*LHX1*), and *LOC102723654* (Table 1). Interestingly, the minor alleles of all the identified SNPs (except for the major allele of rs10048170 *LHX1*) were associated with reduced fatigue parameters [99]. While the exact molecular mechanisms explaining the role of *RTP4*/*MASP2*, and ERN1, SNPs in fatigue-relevant functions and phenotypes are not known, they may be linked through several pathways: 1) the role of *RTP4* in the organisation of γ-δ opioid pain receptors and severe depression and suicidal behaviour during IFNa treatment of hepatitis C infections [110,111]; MASP1 is involved in complement activation through C2 cleavage, thus connecting inflammation and fatigue [112]; ERN1 is a sensor of unfolded proteins in the endoplasmic reticulum, which triggers an intracellular stress signalling pathway known as unfolded protein response [113]. The role of *LHX1* and *LOC102723654* and their SNPs in fatigue-associated regulatory pathways remains to be elucidated.

Novel fatigue-related SNPs were also identified in *BAFF*, another well-known gene associated with pSS. Thus, among five *BAFF* SNPs, only the TT genotype of rs9514828 was less frequent in fatigued compared to non-fatigued Greek pSS patients. Interestingly, no association between the rs9514828 TT genotype and fatigue occurrence was detected in multiple sclerosis patients [100].

Lymphoma: Historically, lymphoma was known to be the most severe complication of SS, as SS patients have a reported 44-fold higher risk of developing lymphoma compared to the general population [114]. Recent meta-analyses, Mendelian randomisation, and Systematic Reviews suggested that non-Hodgkin’s lymphoma (NHL) occurs in approximately 3%–10% of pSS patients, with the most prevalent histological types including mucosa-associated lymphoid tissue (MALT) lymphoma, marginal zone lymphoma (MZL), diffuse large B cell lymphoma (DLBCL), and follicular lymphoma (FL) [115,116,117,118,119]. Further, we describe the recently identified gene variants shown to increase the risk of lymphoma.

Therefore, rs2230926 SNP of *TNF Alpha Induced Protein 3* (*TNFAIP3*), which is involved in the cytokine-mediated immune and inflammatory responses through inhibition of NF-kappa B activation as well as TNF-mediated apoptosis, was shown to enhance the risk of pSS-associated lymphoma in French and UK cohorts. Apparently, rs2230926G variant impaired control of NF-kB activation in B cells that were continuously stimulated by autoimmunity, thus increasing the risk of lymphoma [101]. Subsequently, these results were confirmed in another study conducted on Italian pSS patients [102].

Genetic variations of the *methylene tetrahydrofolate reductase* (*MTHFR*) gene were linked to various cardiovascular diseases, pregnancy complications [120], autoimmune disorders, and lymphoma [121,122]. A recent study characterised rs1801133 and rs1801131 *MTHFR* SNPs as contributors to pSS-related non-MALT lymphomagenesis. In particular, the TT genotype and T allele of rs1801133 and AA genotype and A allele of rs1801131 were more frequent in the pSS non-MALT NHL group compared to controls and patients without NHL. Furthermore, the rs1801133 TT genotype was associated with reduced levels of DNA methylation, while the rs1801131 AC genotype was associated with reduced levels of double-strand breaks. These results suggested that rs1801133 and rs1801131 *MTHFR* variants are involved in the development of pSS-associated non-MALT NHL through their contributions to defective DNA methylation and genomic instability [104].

Genetic variants of *Three Prime Repair Exonuclease 1* (*TREX1*), another gene involved in DNA repair and degradation, have been previously linked to familial chilblain lupus and SLE [123,124]. Recent findings demonstrated a decreased prevalence of rs11797 A allele in pSS patients with non-MALT lymphoma. Furthermore, pSS patients who were carriers of the rs11797 AA genotype showed increased expression of type I IFN-related genes (*Interferon-Induced GTP-Binding Protein Mx1* and *TNF Receptor-Associated Factor 3*) in minor salivary gland tissues. These results suggested a genetically related reduction in type I IFN production as a probable mechanism for pSS-associated lymphomagenesis [105].

Finally, variations of the *Leukocyte immunoglobulin-like receptor A3* (*LILRA3*) gene have been shown to have a drastic effect on both pSS and NHL susceptibility in different populations. Thus, a version with a deletion (absence of the first seven exons) was associated with increased susceptibility to pSS and NHL in the German population [125,126], while in a Chinese population, the functional *LILRA3* variant increased susceptibility to pSS and SLE [127]. Therefore, recent research also linked it to the development of pSS-associated lymphoma in a Greek population, where a functional *LILRA3* gene variant was prevalent in the young pSS patients with lymphoma. Interestingly, young pSS patients with lymphoma showed higher serum LILRA3 protein levels compared to healthy controls [103]. As a possible mechanism, LILRA3 protein may trigger a cellular immune response by activating proliferation of cytotoxic-CD8+T and NK cells [126].

## 5. Conclusions and Future Directions

The role of vitamin D in the pathogenesis and management of primary Sjögren’s syndrome (pSS) remains an area of active investigation with somewhat conflicting findings. While some studies have linked low vitamin D levels to increased pSS severity and activity, others have not observed such an association, suggesting that genetic, environmental, or ethnic factors may play a significant role. Experimental studies in animal models and patient-derived cells have shown that vitamin D supplementation may exert beneficial immunomodulatory effects, such as reducing inflammatory cytokine production, restoring epithelial integrity, and promoting regulatory T cell activity. These findings support the potential of vitamin D application in pSS treatment.

However, the current research has important limitations, including small sample sizes, a lack of ethnic diversity, and inconsistent study designs, which complicate the interpretation of results. Additionally, only one vitamin D-related SNP (VDR rs7975232) has been identified as a potential risk factor for pSS, and its association appears to be specific to certain populations, underscoring the need for further genetic studies.

Future research should aim to explore the immunomodulatory and anti-inflammatory roles of vitamin D more comprehensively. Studies should focus on large, multi-ethnic cohorts to better understand how vitamin D levels and genetic variations, such as VDR polymorphisms, affect disease susceptibility and progression. Investigating the molecular mechanisms by which vitamin D modulates both innate and adaptive immune responses in pSS will be crucial. This includes understanding how vitamin D impacts cytokine production, T and B cell function, and the balance between pro-inflammatory and anti-inflammatory pathways.

Furthermore, clinical trials should evaluate optimal dosing strategies and combinations with other treatments to enhance the therapeutic efficacy of vitamin D. Understanding the gene–environment interactions that influence vitamin D metabolism and its immunomodulatory effects will also provide insights for developing personalised, targeted treatments for pSS, potentially using vitamin D as a core component in managing the inflammatory and autoimmune processes characteristic of the disease.

Looking ahead, integrating genetic testing into clinical practise holds promise for improving disease management and treatment strategies for pSS. Developing comprehensive genetic panels that identify individuals at risk for pSS, its complications, and associated diseases could lead to more targeted interventions. Additionally, investigating gene–environment interactions will provide a better understanding of pSS pathogenesis and inform preventive strategies. Moreover, genetic profiling can assist with predicting disease risk and tailoring treatment strategies. The development of genetic models to assess the risk of pSS based on SNPs, such as those in *STAT4*, *IL-10*, and *HCP5*, could improve disease prediction and management. Personalised approaches to treatment, guided by genetic information, may enhance efficacy and minimise adverse effects.

The development of targeted therapies based on genetic findings offers a new avenue for treatment. Research should focus on drugs or interventions that address specific genetic mechanisms underlying pSS symptoms and complications. Longitudinal studies will be crucial in validating these genetic associations and ensuring that findings translate effectively into clinical practise.

In summary, integrating genetic insights into the understanding and management of pSS offers significant potential for advancing patient care through personalised medicine. By focusing on specific SNPs and their implications for disease risk and severity, future research can lead to more effective and tailored approaches to managing pSS.

## Figures and Tables

**Table 1:** Novel SNPs involved in the development of various pSS-associated diseases.

Gene	SNP	Associated Disease/Mechanism	Patients Notes	References
*OAS1*	rs10774671 A>G	G allele decreased the risk of pSS, anti-SSA-positive SS, and anti-SSA-positive pSS complicated with HBV infection, but not the risk of anti-SSA-negative pSS	Chinese Han pSS patients	[96]
*NUDT15*	rs116855232 C>T	TT genotype was associated with poor enzyme performance, and, subsequently, severe leukopenia after Azathioprine administration	A case study of Chinese pSS patients	[97]
*LINC00243*	rs1264347 C>T	T allele (for rs1264347) and C allele (for rs429150) may facilitate NMOSD pathology	Multiple GWASs’ data	[98]
*TNXB*	rs429150 T>C			
*RTP4/MASP1*	rs60344347 CCTCT>C rs7611640 G>A rs1985269 C>T rs73182503 A>G rs7626469 C>G	Minor allele of these SNPs associated with less fatigue	Swedish and Norwegian cohorts;	[99]
*ERN1*	rs75160892 G>A			
	rs79773538 C>T			
	rs9894399 A>C			
*LHX1*	rs10048170 C>T	C allele associated with less fatigue	Meta-analysis of USA, UK, Swedish and Norwegian cohorts	
*LOC102723654*	rs4509075 T>C	C allele associated with less fatigue		
*BAFF*	rs9514828 C>T	TT genotype associated with lower fatigue levels	Greek and Dutch pSS patients	[100]
*TNFAIP3*	rs2230926 T>G	G allele carriers have more than double the risk of pSS-associated NHL	French and UK cohorts; meta-analysis	[101]
			Of Italian pSS and lymphoma patients	[102]
*LILRA3*	1-7 exon deletion	A functional variant was prevalent in the young pSS patients with NHL; LILRA3 protein serum levels were higher in young pSS patients with NHL	Greek pSS and lymphoma patients	[103]
*MTHFR*	rs1801133 C>T	rs1801133 TT genotype and T allele and rs1801131 AA genotype and A allele were more frequent in the pSS non-MALT NHL group; rs1801133 TT genotype was associated with reduced levels of DNA methylation, while rs1801131 AC genotype with reduced levels of double-strand breaks	Greek pSS and lymphoma patients	[104]
	rs1801131 A>C			
*TREX1*	rs11797 A>G	decreased the prevalence of A allele in pSS patients with non-MALT; pSS patients who were AA genotype carriers showed increased expression of type I IFN-related genes in minor salivary gland tissues	Greek pSS and lymphoma patients	[105]
*THBS1*	rs1478604 C>T	rs1478604 CC and rs2228262 GG carriers showed a higher risk of severe DES development	Russian DES and pSS patients	[106]
	rs2228262 A>G			

*2′–5′ oligoadenylate synthetase* (*OAS1*); *receptor transporter protein 4* (*RTP4)*; Hepatitis B virus (HBV); *Nudix hydrolase 15* (*NUDT15*); *long intergenic non-protein coding RNA 243* (*LINC00243*); *tenascin XB* (*TNXB*); neuromyelitis optica spectrum disorder (NMOSD); *mannan binding lectin serine peptidase 1* (*MASP1*); *endoplasmic reticulum to nucleus signalling 1* (*ERN1*); *LIM homeobox 1 locus* (*LHX1*); *B lymphocyte hyperactivity with B cell activating factor* (*BAFF*); *TNF alpha-induced protein 3* (*TNFAIP3*); *methylene tetrahydrofolate reductase* (*MTHFR*); *mucosa-associated lymphoid tissue* (*MALT*); *three prime repair exonuclease 1* (*TREX1*); *leukocyte immunoglobulin-like receptor A3* (*LILRA3*); *thrombospondin 1* (*THBS1*); dry eye syndrome (DES); non-Hodgkin’s lymphoma (NHL).
